# Effectiveness of Sequential Compression Devices in Prevention of Venous Thromboembolism in Medically Ill Hospitalized Patients: A Retrospective Cohort Study

**DOI:** 10.4274/tjh.galenos.2019.2018.0413

**Published:** 2019-08-02

**Authors:** Prajwal Dhakal, Ling Wang, Joseph Gardiner, Shiva Shrotriya, Mukta Sharma, Supratik Rayamajhi

**Affiliations:** 1 University of Nebraska Medical Center, Department of Internal Medicine, Division of Oncology and Hematology, Omaha, Nebraska, USA; 2 Fred and Pamela Buffett Cancer Center, University of Nebraska Medical Center, Omaha, Nebraska, USA; 3 Michigan State University, Department of Medicine, East Lansing, Michigan, USA; 4 Michigan State University, Department of Epidemiology and Biostatistics, East Lansing, Michigan, USA

**Keywords:** Sequential compression devices, Venous thromboembolism, Hospitalized patients, Effectiveness

## Abstract

**Objective:**

To evaluate the effectiveness of sequential compression devices (SCDs) for venous thromboembolism (VTE) prevention in medically ill hospitalized patients.

**Materials and Methods:**

Adult patients admitted to a teaching hospital from April 2015 to March 2016 were included. Patients on anticoagulants with or without SCDs were excluded. We analyzed VTE risk, length of hospital stay, and other comorbidities among propensity score-matched patients on SCDs and those without thromboprophylaxis (NONE).

**Results:**

Among 30,824 patients, 67 patients (0.22%) developed VTE during their hospital stays, with deep vein thrombosis (DVT) in 55 cases and pulmonary embolism (PE) in 12. VTE was seen in 47 out of 20,018 patients on SCDs (41 DVT, 6 PE) and 20 out of 10,819 patients without SCDs (14 DVT, 6 PE). Risk-adjusted analysis showed no significant difference in VTE incidence in the SCD group compared to NONE (odds ratio 0.99, 95% confidence interval 0.57-1.73, p=0.74).

**Conclusion:**

Compared to the NONE group, SCDs are not associated with decreased VTE incidence during hospital stay.

## Abstract (tr)

**Amaç:**

Çalışmanın amacı hastanede dahili hastalıklar nedeniyle yatan hastalarda ardışık kompresyon cihazlarının (SCD) venöz tromboemboliyi (VTE) önlemedeki etkinliğinin değerlendirilmesidir.

**Gereç ve Yöntemler:**

Çalışmaya Nisan 2015 ile Mart 2016 tarihleri arasında eğitim hastanesine yatan erişkin hastalar alınmıştır. Antikoagülan tedavi alanlar, SCD kullanılsın ya da kullanılmasın, çalışma dışında bırakılmıştır. VTE riski, hastanede yatış süresi ve diğer komorbiditeler eğilim skoru eşlenmiş SCD grubu ve tromboproflaksi kullanmayan grup (NONE) için analiz edilmiştir.

**Bulgular:**

30,824 hastadan 67 hasta (%0,22) hastanede yatış süresi içinde VTE geçirdi, bunların 55 tanesi derin ven trombozu (DVT), 12 tanesi pulmoner emboli (PE) idi. SCD kullanılan 20,018 hastadan 47’sinde (41 DVT, 6 PE), SCD kullanmayan 10,819 hastanın 20’sinde (14 DVT, 6 PE) VTE görüldü. Riske göre düzeltilmiş analiz SCD grubu ve NONE grubu arasında VTE insidansında anlamlı bir farklılık göstermemiştir (odds oranı 0,99, %95 güven aralığı 0,57-1,73, p=0,74).

**Sonuç:**

Yatış süresince SCD grubunda NONE grubu ile karşılaştırıldığında VTE insidansında bir azalma gözlenmemiştir.

## Introduction

Venous thromboembolism (VTE), which includes deep vein thrombosis (DVT) and pulmonary embolism (PE), affects 1 million patients in the United States each year [1,2,3]. Hospitalization is a major risk factor for VTE, with 150-fold increase in risk compared to non-hospitalized individuals [2,4]. Anticoagulants are commonly used for VTE prevention in hospitalized patients, and sequential compression devices (SCDs) are recommended in combination with anticoagulants or when anticoagulants are contraindicated [5]. Current guidelines for SCD use are consensus-based, derived mostly from surgical patients by comparing the effects of SCDs plus anticoagulation versus anticoagulation alone [5,6,7,8]. In routine practice, SCDs are used extensively in hospitals despite limited evidence in medically ill patients [6,9]. We explored the effectiveness of SCDs in medically ill hospitalized patients.

## Materials and Methods

### Participants and Study Design

**Figure 1:**
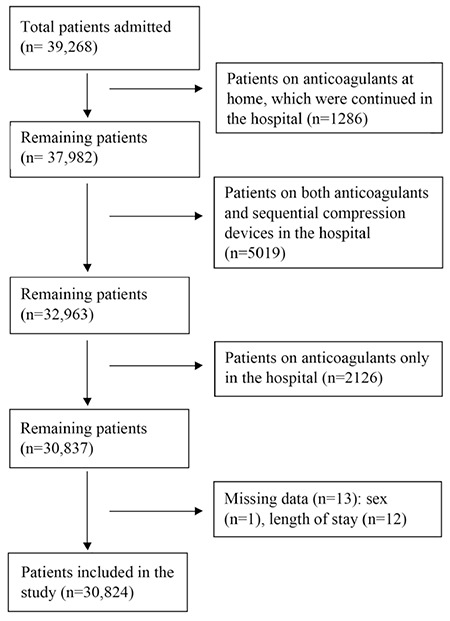
CONSORT diagram for cohort selection.

We included all patients admitted to the medical inpatient service from April 2015 to March 2016 at Sparrow Hospital, a secondary care teaching hospital (Figure 1). Patients <18 years of age or diagnosed with VTE upon admission were excluded. Patients using anticoagulants at home or in the hospital were excluded to eliminate the effects of anticoagulant use. Trained investigators abstracted the data including demographic characteristics, diagnostic methods, methods for VTE prevention, length of hospital stay (LOS), VTE events, and comorbidities. The Charlson Comorbidity Index (CCI) was calculated. Eligible patients were divided into the SCD group (only on SCDs during hospital stay) and the NONE group (no VTE prophylaxis during hospital stay).

### Study Outcomes

The primary outcome was a new diagnosis of symptomatic VTE during the hospital stay. Outcomes were confirmed with Doppler ultrasonography for DVT and computed tomography pulmonary angiogram or ventilation-perfusion scan for PE.

### Statistical Analysis

**Figure 2:**
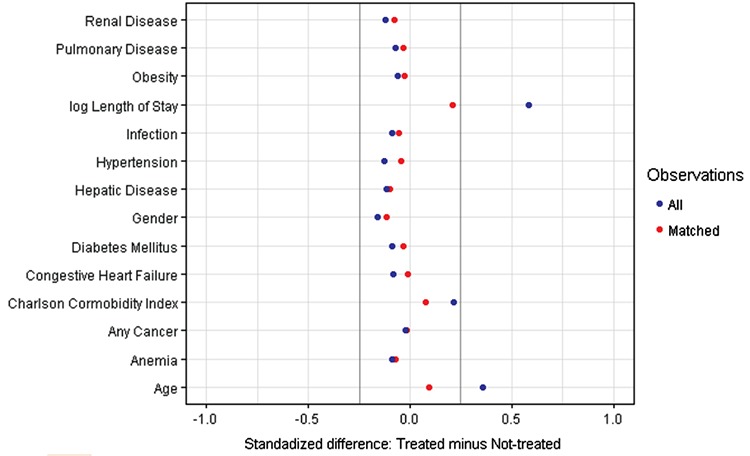
Standardized differences in observed variables between matched pairs. Standardized difference between sequential compression device-treated and matched non-treated patients is the difference in means or proportions divided by an estimate of standard deviation obtained as the square-root of the average variance in treated and non-treated groups. In the matched sample, the differences are within the ±0.25 reference lines for good variable balance.

Differences between the SCD group and NONE group were compared using t-tests or Wilcoxon rank sum tests for continuous variables and chi-square tests for categorical variables. Since patients were not randomly assigned to receive SCDs, propensity score analysis was performed. For each patient, we estimated the propensity score (likelihood of receiving SCD) from a multivariable logistic regression model. There are features of randomness in the selection of treated patients and their matches that could lead to different models for assessing the propensity scores. We experimented with different specifications, especially for LOS and CCI, with the same qualitative conclusion. The variables included in the final model for propensity scores were sex, any type of cancer, comorbidities, and three continuous variables modeled by splines: age (6 terms), log-transformed LOS (3 terms), and CCI (4 terms). A spline function of a continuous variable is a smooth function composed of polynomial pieces connected at interior points called knots in the range of the variable [10,11]. The c-statistic was 0.707, indicating an acceptable level of discrimination between SCD and NONE patients. Figure 2 depicts the adjusted odds ratios (ORs) and 95% confidence intervals (CIs) for the binary variables. We followed published principles and guidelines to form treated and non-treated pairs based on their propensity scores [12,13]. A randomly chosen SCD patient was matched to one NONE patient in the common region of propensity scores extended by 0.25 times the pooled estimate of the standard deviation of the logits of propensity scores in the two groups. This greedy matching algorithm, which proceeded sequentially with SCD patients selected in random order of propensity scores and matched to a unique NONE patient, resulted in 10,071 unique pairs. The SAS procedure PSMATCH was used for matching. In the matched sample, we examined the quality of the matching by comparing the standardized mean differences and variance ratios between SCD and NONE [14,15]. We used conditional logistic regression to obtain the adjusted OR and 95% CI for the association of SCDs with VTE incidence. We also performed a risk-adjusted analysis for VTE incidence with an indicator of SCD use. A multivariable logistic model with a subset of the covariate mix was applied using information criteria for model selection [16].

The study was determined exempt by Michigan State University and Sparrow Hospital with IRB # i051275.

## Results

### Patient Characteristics

A total of 30,824 patients were included in the analysis; mean age was 54±21 years and 61.5% were female. Mean CCI was 4.5±2.4. Mean LOS was 4.5±4.3 days. Out of the total patients, 20,018 (64.9%) were on SCDs and 10,819 (35.1%) were not. Patient characteristics, including those on anticoagulants, are provided in Table 1.

### Outcome

Sixty-seven (0.22%) patients had VTE, with DVT in 55 cases and PE in 12 cases. DVT and PE occurrences in the SCD group were 41 and 6, compared to 14 and 6 in the NONE group. Thus, 0.23% of total patients on SCDs developed VTE compared to 0.18% in the NONE group.

### SCD Impact on VTE Incidence

In the unadjusted analysis, use of SCDs was not associated with decreased VTE incidence (OR 1.27, 95% CI 0.75-2.14, p=0.37) (Table 2). Conditional logistic regression after propensity matching yielded an adjusted OR of 0.9 (95% CI 0.47-1.7, p=0.75) for VTE incidence with SCDs. Similarly, the adjusted OR for SCDs after multivariable logistic regression was 0.99 (95% CI: 0.57-1.73, p=0.98).

## Discussion

Our large retrospective study of 30,824 medically ill patients demonstrated a similar incidence of VTE with SCDs only compared to the NONE group. In comparison to NONE, SCD patients had significant differences in risk factors for VTE, including higher CCI, higher prevalence of cancer and obesity, and longer LOS. Propensity score matching matched the SCD and NONE groups with no statistical difference in VTE incidence. The overall incidence of symptomatic VTE was <1% in our study, which might have played a role in the results. Previous studies reported significantly higher incidences of VTE in critically ill patients compared to other non-critical medically ill patients [17,18,19,20,21]. Critically ill patients on anticoagulants or both anticoagulants and SCDs were not eligible for analysis in our study, which could be one of the causes of the lower incidence of VTE. Multiple studies also screened patients for VTE before discharge, which would lead to the diagnosis of asymptomatic VTE and subsequently increase the overall incidence of VTE [22]. We studied symptomatic patients only and no screening for asymptomatic VTE was performed.

Despite significant results in surgical patients, the existing literature contains mixed results regarding the use of SCDs in medically ill patients. This could be related to publication bias in these types of study. Limpus et al. [23] performed a systematic review of compression and pneumatic devices for thromboprophylaxis in intensive care patients. Twenty-one studies with >4000 individuals were analyzed and there was no significant difference with the use of compressive and pneumatic devices compared to no treatment or use of anticoagulants [23]. In another review, the strength of the evidence was insufficient to determine the effectiveness of SCDs for thromboprophylaxis in high-risk medical patients because of limited data [24]. The CLOTS III trial reported significant effectiveness of SCDs in DVT prevention in immobile patients with acute stroke. Since these patients were considerably less mobile, the results may not be reproducible in our study. Some other studies reported lower incidence of VTE with SCDs compared to NONE but the results were not statistically significant [7,25].

Our study should be viewed in the context of its strengths and limitations. Although the risk of VTE in hospitalized patients tends to persist for weeks after hospitalization, we focused on VTE during hospital stay, which might have led to decreased VTE incidence [26]. In fact, the number of symptomatic VTE events during hospital stay in medically ill patients has been reported to be similar to the number of VTE events after discharge [26]. However, with VTE incidence of <1%, the projected number of VTE events after discharge in our study population would still be lower than that reported in the literature. Our analysis excluded high-risk patients who received anticoagulants with or without SCDs and thus may not represent all hospitalized medical patients seen in clinical practice. The compliance and appropriate use of the SCDs could not be verified in all cases. However, this is one of the few analyses looking at the effectiveness of SCDs in acutely medically ill patients. We matched patients from a large sample to minimize many potential confounders of association between the preventive methods and outcomes. The number of patients given anticoagulants was modest and lower than recommended by many contemporary guidelines. Our study supports scaling back the current guidelines recommending widespread use of anticoagulants or SCDs until better prospective evidence from randomized trials is available.

## Conclusion

Compared to the NONE group, SCD usage was not associated with decreased VTE incidence. VTE incidence was <1% during hospital stay, although asymptomatic VTE may have occurred before discharge. The strength of the evidence might be insufficient to exclude clinically important differences in treatment effects because of selection bias in the choice of therapy, undetermined number of VTE events after discharge, and exclusion of higher-risk patients on anticoagulation. Further prospective studies are needed to clarify the role of SCD in medically ill patients.

## Figures and Tables

**Table 1:**
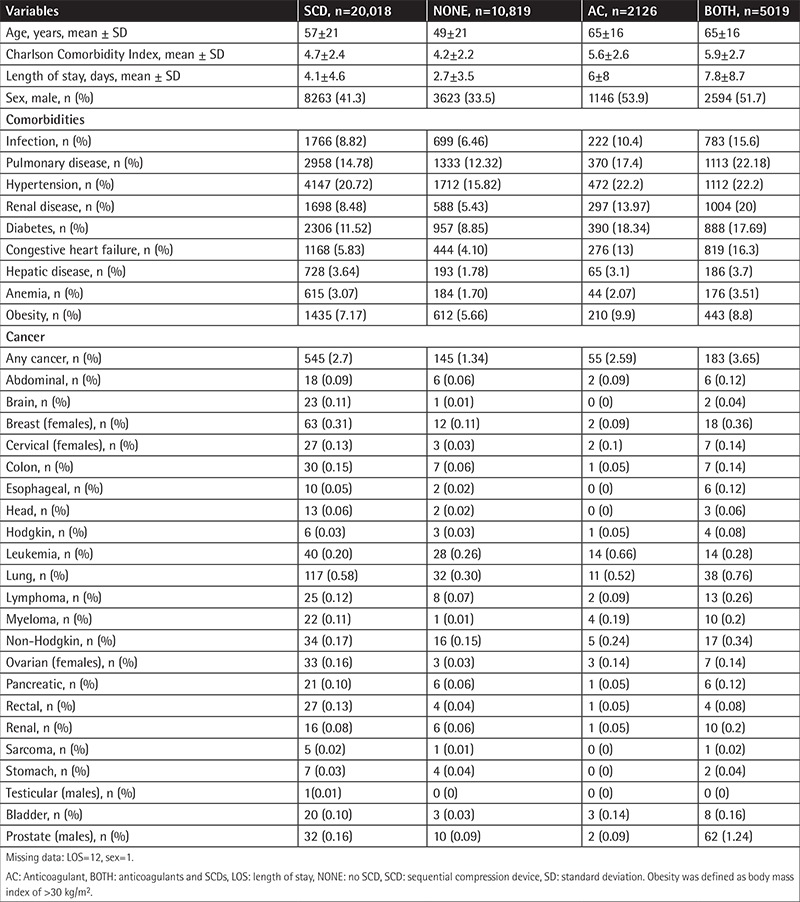
Patient characteristics.

**Table 2:**
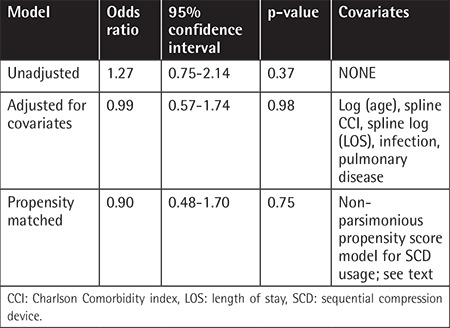
Effect of sequential compression devices on incidence of venous thromboembolism compared to no prophylaxis.
